# Use of Probiotics with Antibiotics: Knowledge, Attitudes, and Practices in Community Pharmacy

**DOI:** 10.3390/healthcare14162602

**Published:** 2026-08-19

**Authors:** Zorica Naumovska, Beti Djurdjic, Kristina Mladenovska, Emilija Atanasovska, Tanja Petreska Ivanovska, Teodora Tasevska, Katarina Stavrikj, Biljana Bozhinovska, Joana Andonoska, Katarina Smilkov, Maja Simonoska Crcarevska

**Affiliations:** 1 Faculty of Pharmacy, Ss. Cyril and Methodius University in Skopje, 1000 Skopje, North Macedonia; krml@ff.ukim.edu.mk (K.M.); tpetreska@ff.ukim.edu.mk (T.P.I.); t.tasevska@ff.ukim.edu.mk (T.T.); 2 Research Center for Excellence in Education and Science, 81000 Podgorica, Montenegro; beti.djurdjic@gmail.com; 3 Faculty of Medicine, Ss. Cyril and Methodius University in Skopje, 1000 Skopje, North Macedonia; emilija.atanasovska@medf.ukim.edu.mk (E.A.); kstavric@hotmail.com (K.S.); 4 The Association of Private Community Pharmacies of the Republic of North Macedonia, 1000 Skopje, North Macedonia; bile@annifarm.com.mk (B.B.); joana.andonoska@hotmail.com (J.A.); 5 Faculty of Medical Sciences, Goce Delcev University, 2000 Stip, North Macedonia; katarina.smilkov@ugd.edu.mk

**Keywords:** community pharmacy, counseling, antibiotic-associated diarrhea, probiotics, continuing professional education

## Abstract

**Highlights:**

**What are the main findings?**

- Community pharmacy professionals in North Macedonia demonstrated generally moderate-to-high alignment with the prespecified survey scoring key, positive attitudes, and frequent self-reported recommendation practices regarding probiotics use for the prevention of antibiotic-associated diarrhea.
- Lower alignment with the prespecified survey scoring key was identified for several clinical aspects of probiotic use, including whether probiotics should be administered with food, the recommended duration of use following antibiotic therapy, and their safety in immunocompromised patients.

**What are the implications of the main findings?**

- Continuous professional education was associated with stronger self-reported counseling practices and targeted continuing education may therefore be considered to support more evidence-based individualized recommendations regarding probiotic use.
- Strong support for structured counseling services and national probiotic guidelines provides a basis for considering nationally adapted educational resources, counseling frameworks and evidence-based guidelines, which should be prospectively evaluated before conclusions are drawn regarding their effects on practice or patient outcomes.

**Abstract:**

Background: Antibiotic-associated diarrhea (AAD) is a common consequence of gut microbiota disruption following broad-spectrum antibiotic use. Certain probiotics may reduce the incidence of AAD. However, their effectiveness also depends on appropriate counseling by community pharmacy professionals and implementation of evidence-based practice. Objectives: To assess the knowledge, attitudes, and practices (KAP) of community pharmacy professionals in North Macedonia regarding concomitant antibiotic–probiotic use and to identify factors associated with these domains. Methods: An anonymous content-validated online questionnaire was distributed to a convenience sample of community pharmacists and pharmacy technicians in North Macedonia (April–May 2026). Data were analyzed using descriptive statistics, chi-square tests, factor analysis, and regression models in Jamovi (version 2.6) and R software (version 4.4). A *p*-value <0.05 was considered statistically significant. Results: Among 285 respondents (mean age 41.8 ± 9.6 years), 54.7% were classified in the high category of the survey-based knowledge index according to the prespecified survey key. Recognition of probiotic efficacy was high (95.8%), but alignment with the prespecified survey key regarding proper administration with food and risk identification was lower (34.6% and 66.8%, respectively). Attitudes toward probiotic use were highly positive, and recommendation practices were frequently reported (84.5%), although counseling practices varied. More positive attitudes were associated with higher recommendation behavior (β = 0.512, *p* < 0.001) and female gender was also associated with higher scores (*p* = 0.008). Recent continuous professional education was associated with higher counseling-related practice scores (*p* < 0.001), while greater variability in counseling behavior was observed across community pharmacy professionals. Conclusions: Community pharmacy professionals in North Macedonia demonstrated favorable attitudes and frequent self-reported recommendation practices regarding probiotic use alongside antibiotics. However, important gaps remain in clinical knowledge and counseling consistency. Targeted continuing professional education and development of standardized national guidelines may be considered, but their effects on observed practice and patient outcomes require prospective evaluation.

## 1. Introduction

Antibiotics remain among the most commonly prescribed medicines worldwide, with global consumption exceeding 30 billion defined daily doses annually, reflecting a sustained increasing trend over recent decades [1,2]. However, a substantial proportion of antibiotic use is inappropriate, with estimates ranging from 30% to 60% of prescriptions being unnecessary, suboptimal, or incorrectly selected [3]. This inappropriate use contributes not only to antimicrobial resistance but also to a significant burden of preventable adverse drug reactions, increased healthcare utilization, and avoidable economic costs.

Antibiotic-associated diarrhea (AAD) is one of the most common adverse effects of antibiotic therapy, occurring in approximately 5–35% of treated patients and typically developing during treatment or up to 8 weeks after antibiotic discontinuation [4]. Although often mild and self-limiting, AAD is clinically relevant, as it may lead to treatment discontinuation, additional diagnostic procedures and increased healthcare use. Importantly, up to one-third of cases are associated with *Clostridium difficile* infection (CDI), which is linked to prolonged hospital stay, recurrent admissions, increased morbidity and substantial healthcare costs, thereby contributing significantly to overall system burden. In Europe, approximately 124,000 cases occur annually and CDI is ranked as the sixth most frequently detected healthcare-associated infection in the European Prevalence Study (2016–2017) [5,6]. Community-associated CDI further adds an estimated incidence of 51.9 cases per 100,000 population. In the United States, CDI accounts for approximately 453,000 cases and 29,000 deaths annually, corresponding to a mortality rate of around 6.4% [7]. These data highlight the substantial global clinical and economic burden of antibiotic-associated complications.

Overall, AAD and CDI are primarily driven by antibiotic-induced disruption of the intestinal microbiota, which increases susceptibility to gastrointestinal morbidity and treatment-related complications. From a healthcare system perspective, these conditions contribute to increased hospital length of stay, recurrent admissions, additional diagnostic procedures and higher treatment costs, particularly in severe or recurrent cases. This underscores the need for effective preventive strategies aimed at preserving gut microbiota integrity during antibiotic therapy.

Global clinical evidence, including findings from extensive meta-analyses and systematic reviews, indicates that the concomitant use of specific probiotic strains or well-defined combinations administered at adequate doses can significantly reduce the incidence and severity of AAD in certain populations by supporting the restoration of beneficial microflora and intestinal homeostasis [8,9,10,11,12,13]. Probiotics containing specific strains of *Lactobacillus*, *Bifidobacterium*, and *Saccharomyces* have been shown to reduce the incidence of AAD, particularly when probiotic supplementation is initiated early during antibiotic therapy. Because patients frequently receive antibiotics and probiotics simultaneously at the point of dispensing, healthcare providers serve as critical gatekeepers in supporting their safe and evidence-based use [11]. As the most accessible frontline healthcare professionals, community pharmacy professionals play a pivotal role in antimicrobial stewardship and public health promotion. They are responsible for auditing prescriptions, preventing inappropriate antibiotic use and providing evidence-based patient counseling. Within the context of antibiotic–probiotic co-administration, community pharmacy professionals are well positioned to optimize therapy through evidence-based counseling on appropriate strain selection, dosing regimens and administration timing with the aim of minimizing potential interactions and maximizing probiotic effectiveness.

Importantly, the community pharmacy professionals bear the responsibility of addressing patient misconceptions, minimizing inappropriate self-medication behaviors and preventing premature discontinuation of antibiotic therapy. They also contribute to patient safety by identifying individuals at increased risk who may not benefit from probiotic use or who may require referral to a healthcare professional. Without accurate and consistent counseling, the therapeutic potential of probiotics may be compromised, leading to suboptimal outcomes and preventable gastrointestinal disturbances. The quality of such counseling is highly dependent on community pharmacy professionals’ knowledge base, attitudes, and routine dispensing practices.

Recent international literature highlights persistent gaps in community pharmacy professionals’ understanding of probiotic strains, mechanisms of action, storage conditions and evidence-based clinical indications. Although they generally demonstrate positive attitudes toward probiotics, studies report limited knowledge regarding strain-specific efficacy, non-gastrointestinal applications and optimal use in antibiotic-associated conditions. This knowledge–practice gap often results in inconsistent counseling and suboptimal integration of probiotics into routine clinical practice [11,14,15,16,17,18].

In North Macedonia, increasing availability of probiotics as over-the-counter products, combined with widespread antibiotic use, underscores the need for a systematic evaluation of community pharmacy professionals-led counseling practices. Variability in knowledge and absence of standardized national guidelines may contribute to inconsistent recommendations and suboptimal patient outcomes. Despite growing global evidence on healthcare providers’ perspectives regarding probiotics, the lack of country-specific data reflecting the practices of community pharmacists and pharmacy technicians in North Macedonia persists. Therefore, this study aims to assess the knowledge, attitudes, and practices (KAP) of community pharmacists and pharmacy technicians regarding the co-administration of antibiotics and probiotics using a cross-sectional questionnaire-based design, with the goal of identifying areas of uncertainty and variability that may inform future educational and policy considerations.

## 2. Materials and Methods

### 2.1. Study Design and Setting

A cross-sectional, questionnaire-based survey was conducted among community pharmacy professionals (community pharmacists and pharmacy technicians) in North Macedonia to assess their knowledge, attitudes, and practices (KAP) regarding the combined use of antibiotics and probiotics. Both community pharmacists and pharmacy technicians were included because both professional groups are involved in supply of probiotic products and communication with patients about their use in community pharmacy practice in North Macedonia. The study employed an anonymous, self-administered online questionnaire.

### 2.2. Questionnaire Development and Validation

Data were collected using a structured questionnaire specifically developed for this study, comprising four sections: demographic and professional characteristics, knowledge, attitudes, and practices related to probiotic use in AAD. The instrument was developed based on the relevant literature, including systematic reviews and clinical guidelines.

During questionnaire development and before data collection, the item-level response key for Q7–Q15 was prespecified based on the available evidence and guidance [8,9,10,12,19,20]. Responses concerning general evidence, mechanisms, and effects (Q7–Q9), administration, treatment initiation and duration (Q10–Q13), safety (Q14), and fixed-dose product availability (Q15) were treated as survey-specific, evidence-aligned scoring benchmarks rather than universal clinical rules, because probiotic use depends on the strain or product, dose, patient population, indication, and clinical context. Q14 identified a prespecified high-risk group rather than providing an exhaustive safety classification, while Q15 reflected product availability in North Macedonia at that time. Q16–Q22 assessed attitudes and related perceptions, whereas Q23–Q29 assessed self-reported practices. Unlike the knowledge items, these responses were not evaluated against the prespecified survey scoring key. Where composite scores were constructed, responses were coded according to favorability (Q16–Q18) or reported frequency (practice items), rather than interpreted as direct measures of guideline-concordant care. Neither the questionnaire items nor the survey scoring key was changed after data collection.

Face validity was assessed by three academic experts in clinical pharmacy, pharmacoinformatics, community pharmacy practice, and pharmaceutical technology, who evaluated clarity, wording, and appropriateness for the target population prior to content validation.

Content validity was evaluated by a multidisciplinary panel of eight experts from clinical pharmacy, family medicine, pharmacology, pharmaceutical technology and biotechnology, food science and nutrition, and community pharmacy practice. Each item was rated for relevance using a 4-point scale (1 = not relevant to 4 = highly relevant). The Item-level Content Validity Index (I-CVI) was calculated as the proportion of experts rating each item as 3 or 4.

At the scale level, the Scale-level Content Validity Index (S-CVI/Ave) and the Scale-level Content Validity Index based on universal agreement (S-CVI/UA) were calculated to assess the overall content validity. A priori thresholds of ≥0.78 for I-CVI and ≥0.90 for S-CVI/Ave were considered indicative of acceptable and excellent validity, respectively [21,22]. The Modified Kappa statistic (κ*) was used to adjust for chance agreement.

### 2.3. Data Collection

The questionnaire was distributed electronically via Google Forms. The survey link was disseminated through the Association of Private Pharmacies in North Macedonia and shared within closed professional groups on social networks. Data collection took place between 28 April and 20 May 2026. Eligible participants included licensed community pharmacists and pharmacy technicians actively working in community pharmacies in North Macedonia. Given that recruitment was conducted through professional networks and the survey link could be further shared, a convenience sampling approach was used, whereby all eligible professionals who completed the survey during the study period were included. Therefore, no formal a priori sample-size calculation was performed. Moreover, the number of eligible professionals who received or viewed the survey invitation could not be determined, precluding the calculation of a conventional response rate. Participation was voluntary, anonymous, and without incentives. No personal identifiers were collected. Participants were informed about the study objectives, approximate completion time (5–7 min), and the exclusive use of data for scientific purposes. The questionnaire comprised a total of 29 items, of which all except three items (Q14, Q20, and Q24) were single-choice questions.

### 2.4. Statistical Analysis

Data were analyzed using descriptive and inferential statistical methods in Jamovi (version 2.6) [23], with additional analyses performed using R (version 4.4) [24] within Jamovi. Categorical variables were presented as frequencies and percentages. Descriptive and item-level analyses were based on the available responses for each item. Associations between variables were initially assessed using Pearson’s chi-square test. The distribution of expected cell counts was examined for each contingency table. Pearson’s chi-square test was retained when no expected cell count was <1 and no more than 20% of expected cell counts were <5. When these assumptions were not met, Fisher’s exact test was used for 2 × 2 tables, while the Fisher–Freeman–Halton exact test with Monte Carlo simulation (B = 200,000) was used for larger contingency tables. Effect sizes were reported using Cramer’s V. Their magnitude was interpreted according to the dimensions of each contingency table, using k = min(r − 1, c − 1), where r is number of rows, and c is number of columns [25]. The conventional thresholds for small, medium, and large effects were calculated as (0.10/sqrt(k)), (0.30/sqrt(k)) and (0.50/sqrt(k)), respectively [25,26]. These thresholds were treated as descriptive benchmarks and interpreted together with the observed response distributions. For ordinal variables, Kendall’s tau-b for the linear trend was applied where appropriate. To account for multiple comparisons, *p*-values from the appropriate tests were adjusted collectively using the Benjamini–Hochberg false discovery rate (FDR) procedure. An FDR-adjusted *p*-value < 0.05 was considered statistically significant.

### 2.5. Score Construction

Composite scores were constructed prior to exploratory factor analysis using a prespecified scoring approach. The survey based knowledge index and attitude scores were derived using a dichotomized approach to improve interpretability and comparability across domains. The knowledge index was calculated as the sum of responses to Q7–Q15 that matched the prespecified response key (Q14 was included despite its multiple-response format because it assessed recognition of predefined high risk patient groups and could be converted into a single dichotomous outcome using a prespecified criterion). Responses were coded as 1 for matching the survey scoring key and 0 when they did not match or respondents were uncertain. For Q14, one point was assigned only when the prespecified survey key option was selected and all other response patterns received zero points. The knowledge index yielded a range of 0–9 and were categorized as low (0–3), moderate (4–6), and high (7–9). Candidate items for the attitude domain comprised Q16–Q22, excluding Q20 because it was a multiple-response item. These items were initially evaluated using EFA. Based on the resulting factor structure, the final attitude score was calculated from Q16–Q18 only, which formed a coherent construct. Responses reflecting a favorable attitude were coded as 1, whereas all other responses were coded as 0, yielding a possible score range of 0–3. Q19, Q21, and Q22 were excluded from the final score and analyzed separately as innovation-related perceptions. Practice variables (Q23–Q29, excluding Q24) were assessed using Likert-type frequency scales (4 and 5-point), with higher scores indicating more frequent self-reported clinical practice. Q20 and Q24 were excluded because their multiple-response formats could not be reduced to a single meaningful score.

### 2.6. Exploratory Factor Analysis (EFA)

Exploratory factor analysis (EFA) was conducted to assess the construct validity of the knowledge, attitude, and practice domains. Sampling adequacy was evaluated using the Kaiser–Meyer–Olkin (KMO) measure, and Bartlett’s test of sphericity confirmed the suitability of the data for factor analysis. The minimum residual extraction method with oblimin rotation was applied to allow for potential correlations between factors. The number of factors was determined based on eigenvalues greater than 1 and visual inspection of the scree plot. Items with factor loadings ≥ 0.40 were considered meaningful for interpretation. KMO values ≥ 0.60 were considered acceptable for factorability, whereas values close to this threshold were considered borderline and interpreted cautiously. To further evaluate the two-factor practice structure identified by EFA, a post hoc confirmatory factor analysis was performed using diagonally weighted least squares (DWLS) estimation with robust standard errors for ordinal indicators. Model fit was evaluated using the scaled χ^2^ test, CFI, TLI, RMSEA with 95% confidence intervals, and SRMR. The results of the EFA were used to guide the derivation of subscale scores used for subsequent analyses, with subscales derived from borderline factor solutions treated as exploratory and further evaluated using post hoc confirmatory factor analysis (CFA). Because the CFA was post hoc and used the same sample, it was considered an internal structural check rather than independent confirmation.

### 2.7. Regression Analysis

Multiple linear regression analyses were conducted to examine factors associated with attitudes (Q16–Q18), recommendation-related practice (Q23, Q25, Q29), and counseling-related practice (Q26–Q28). The attitude model included sociodemographic characteristics (gender, age, years of experience, pharmacy type, professional position, and prior education on probiotics). The recommendation and counseling-related practice models included the same variables, with the attitude score additionally entered as an explanatory variable. The total knowledge index was not included because the knowledge items did not support a reliable unidimensional scale. All models were estimated using the ordinary least squares (OLS) method with listwise deletion for missing data. Model fit was assessed using R^2^ and adjusted R^2^. Standard regression assumptions were evaluated, including multicollinearity (VIF < 5), residual independence (Durbin–Watson), normality (Shapiro–Wilk and Q–Q plots), and influential observations (Cook’s distance). To assess the robustness of the findings to violations of regression assumptions, the final linear regression models were re-estimated using the GAMLj module in Jamovi (version 2.6) with HC3 robust standard errors and non-parametric percentile bootstrap 95% confidence intervals based on 5000 resamples. Analyses were performed in Jamovi (version 2.6), and R (version 4.4) with *p* < 0.05 was considered statistically significant [23,24].

Ordinal logistic regression (cumulative link model with logit link) was used for innovation-related outcomes (Q19, Q21). Q22 was assessed for modeling but was not estimated because near-unanimous responses provided insufficient outcome variability. Sociodemographic variables, together with the attitude, recommendation and counseling related practice scores, were included as explanatory variables. Model performance was assessed using likelihood ratio χ^2^ tests and pseudo-R^2^, and results were reported as coefficients (β) and odds ratios (OR) with 95% confidence intervals. Model diagnostics included convergence and stability checks. Listwise deletion was applied, and statistical significance was set at *p* < 0.05.

Missing-data patterns for variables included in the three final linear regression models and the ordinal regression models for Q19 and Q21 were examined using Little’s MCAR test. Q22 was excluded from this model-specific assessment because its near-unanimous response distribution did not permit stable ordinal regression estimation and it was therefore only analyzed descriptively. As a sensitivity analysis, multiple imputation by chained equations (MICE) was performed using the mice package in R. Twenty imputed datasets were generated over 20 iterations. Predictive mean matching was used for age and years of professional experience, binary logistic regression for gender and professional position, polytomous logistic regression for pharmacy type and proportional-odds regression for the ordinal Q19 outcome. Q21 had no missing observations and therefore did not require imputation but was retained in the imputation and analysis dataset. All variables included in the three final regression models were incorporated into the imputation model. The final linear and ordinal regression models were re-estimated in each imputed dataset and the estimates were pooled using Rubin’s rules.

To reduce the risk of model overfitting, the final models were restricted to theoretically relevant variables, while predictors based on psychometrically unsupported composite scores were excluded. Sensitivity power analysis was performed using PAMLj in Jamovi (version 2.6) to estimate the minimum detectable overall effect sizes for the final linear regression models, assuming *N* = 260, α = 0.05, and 80% power. No formal power analysis was performed for the ordinal logistic regression models because power depends strongly on the distribution of outcome categories and predictor structure.

## 3. Results

### 3.1. Content Validity

Face validity confirmed that the questionnaire items were clear, understandable, and appropriate for the target population, with minor linguistic refinements made prior to content validation. I-CVI values ranged from 0.75 to 1.00, with the majority of items (23/29) achieving perfect agreement (I-CVI = 1.00). The S-CVI/Ave was 0.966, indicating excellent overall content validity, while S-CVI/UA was 0.793, reflecting substantial but more conservative agreement among experts. Modified kappa values ranged from 0.70 to 1.00, confirming good to excellent agreement beyond chance. Overall, these results demonstrate strong content validity and support the appropriateness of the instrument for the intended study population and objectives.

### 3.2. Exploratory Factor Analysis

EFA was conducted to assess construct validity of the knowledge, attitude, and practice domains. Knowledge (Q7–Q15), attitude (Q16–Q22), and practice (Q23–Q29) items were included, excluding multiple-response items (Q20, Q24). For the knowledge domain sampling adequacy was borderline (KMO = 0.592), while Bartlett’s test was significant (*p* < 0.001). EFA did not support a unidimensional structure (Eigenvalue < 1), with weak loadings and high uniqueness values, indicating heterogeneous content. Therefore, knowledge was treated as a formative construct and analyzed primarily at item level. Exploratory thematic groupings also showed poor internal consistency (Cronbach’s α = 0.126 for Q7–Q9, 0.396 for Q10–Q13 and 0.110 for Q14–Q15; overall nine-item index α = 0.408); therefore, no thematic knowledge subscales were constructed. For the attitude domain, sampling adequacy was acceptable (KMO = 0.672) and Bartlett’s test was significant (*p* < 0.001). A single dominant factor was identified (Eigenvalue = 1.55; 25.7% variance). Items Q16–Q18 loaded strongly and formed a coherent scale (Cronbach’s α = 0.736), while Q19, Q21, and Q22 showed weak or unstable loadings and were excluded from the final attitude score and analyzed separately as innovation-related perceptions. For the practice domain, KMO indicated borderline adequacy (KMO = 0.591) with a significant Bartlett’s test (*p* < 0.001) and therefore, the resulting factor structure should be interpreted cautiously. Two interpretable factors emerged—recommendation-related practice (Q23, Q25, Q29) and counseling-related practice (Q26–Q28)—explaining 26.9% of variance for the primary factor. Although the second factor did not meet the eigenvalue criterion, it was retained due to theoretical interpretability. Internal consistency was acceptable for the recommendation-related practice subscale (Cronbach’s = 0.703), but lower for the counseling-related practice subscale (Cronbach’s = 0.613) indicating limited scale reliability. A post hoc confirmatory factor analysis was subsequently conducted to further evaluate the proposed two-factor practice structure. The DWLS model with robust standard errors for ordinal indicators showed good fit: scaled χ^2^(8) = 5.03, *p* = 0.755, CFI = 1.000, TLI = 1.00, scaled RMSEA = 0.000 (95% CI: 0.000–0.049), and SRMR = 0.032. Standardized factor loadings ranged from 0.649 to 0.976 for recommendation-related practice and from 0.532 to 0.794 for counseling-related practice. The correlation between the two factors was weak and not statistically significant (r = 0.140, *p* = 0.167). The post hoc CFA provided additional support for the theoretically defined two-factor practice structure. Given the lower reliability of the counseling-related practice subscale, findings involving this subscale were interpreted cautiously.

### 3.3. Participant Characteristics

A total of 285 respondents participated in the study. The number of valid responses varied across questionnaire items because of item-level nonresponse. Missingness was low, ranging from 0 to 12 responses per item (0.0–4.2%), as presented in Supplementary Table S1. Among respondents with available data, the mean age was 41.8 years (SD = 9.63; range 23–68), and the mean work experience was 17.0 years (SD = 9.6; range 0.5–44). Female respondents predominated in the sample (88.4%, 252/285). The main demographic and professional characteristics of the participants, including community pharmacy type, professional position, and participation in continuous education on probiotics, are presented in Table 1. The majority of respondents worked in large pharmacy chains (57.9%, 165/285) and were pharmacists (66%, 188/285), while 60.4% (172/285) reported having attended continuous education on probiotics within the last two years.

Among respondents with complete data on gender and professional position, 164 of 185 pharmacists were women (88.6%) and 21 were men (11.4%). This distribution closely matched the national register of pharmacists, in which 1310 of 1480 registered pharmacists were women (88.5%) and 170 were men (11.5%). A one-sample chi-square goodness-of-fit test showed no statistically significant difference between the sample and national sex distributions, χ^2^(1) = 0.003, *p* = 0.954. Among pharmacy technicians, 87 of 95 respondents were women (91.6%) and eight were men (8.4%). However, comparable national gender-related data for pharmacy technicians were unavailable.

Using an official extract of the publicly available national pharmacy register supplied by MALMED, 1030 active regular community pharmacy premises were identified [27]. Premises were grouped by operator primarily according to tax identification number, with the company registration number used where necessary. The resulting distribution comprised 411 independent pharmacies (39.9%), 210 premises belonging to small chains (20.4%), 113 to medium-sized chains (11.0%), and 296 to large chains (28.7%). In the study sample with available data on pharmacy type (*n* = 281), 19.9% of respondents worked in independent pharmacies, 11.7% in small chains, 9.6% in medium-sized chains, and 58.7% in large chains. Thus, compared with the distribution of community pharmacy premises, respondents employed in large chains were more frequently represented, whereas those from independent pharmacies and small chains were less frequently represented. However, the nationally available data refer to pharmacy premises rather than the number of employed individual pharmacists and pharmacy technicians and therefore cannot be interpreted as the population distribution of pharmacy personnel by workplace type.

### 3.4. Community Pharmacy Professionals’ Responses to Knowledge Items Regarding Probiotics and Antibiotic-Associated Diarrhea

**Figure 1:**
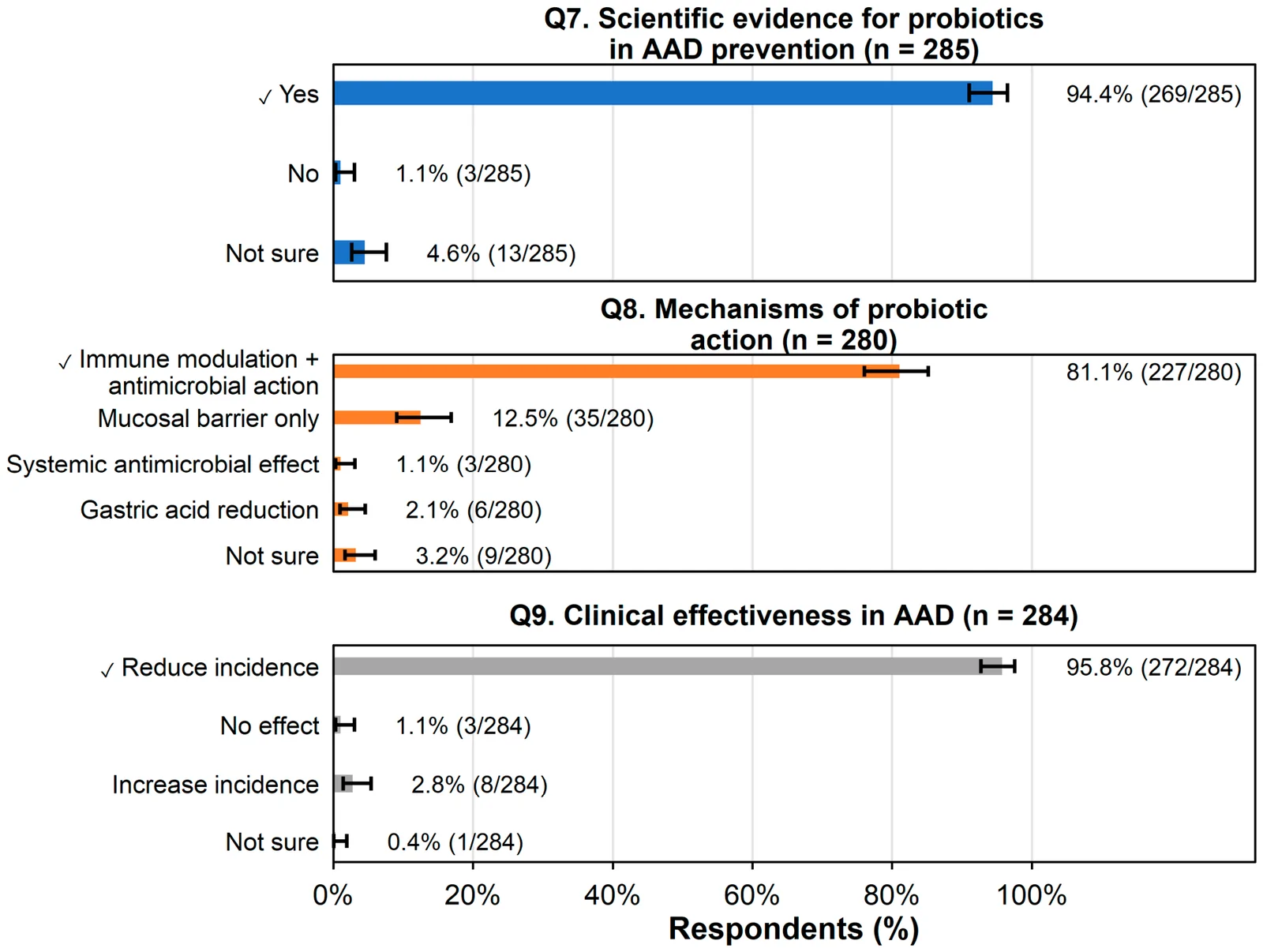
Community pharmacy professionals’ responses to knowledge items regarding evidence, mechanisms of probiotic use and clinical effectiveness of probiotics in AAD (Q7–Q9), where ✓ indicates prespecified evidence-aligned response used in the survey scoring key. Bars represent percentages of valid responses and error bars indicate 95% Wilson confidence intervals.

**Figure 2:**
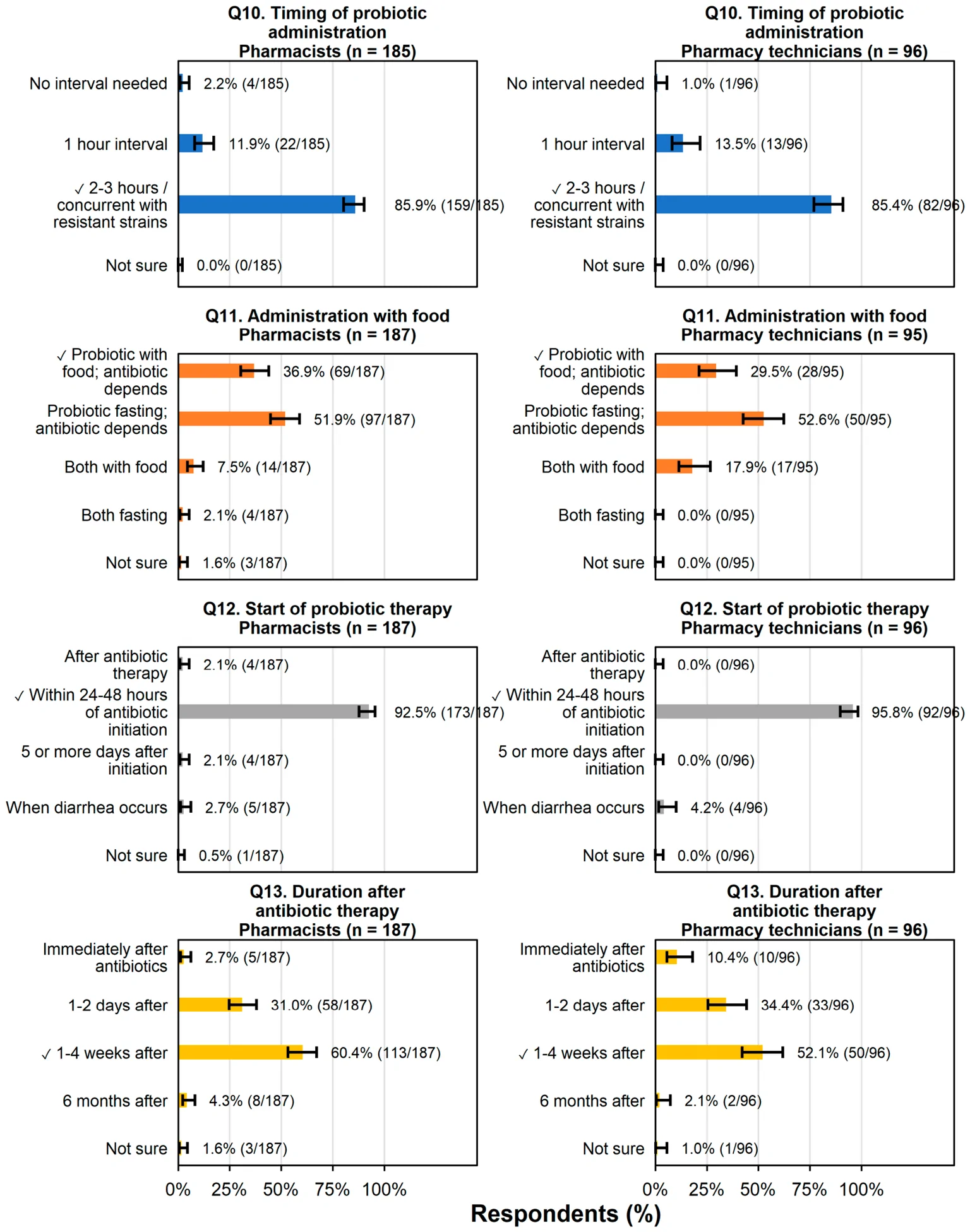
Community pharmacy professionals (pharmacists’ and pharmacy technicians’) responses to knowledge items regarding the use of probiotics with antibiotics, focusing on clinical use and administration (Q10–Q13) stratified by professional position, where ✓ indicates the prespecified evidence-aligned response used in the survey scoring key. Bars represent percentages of valid responses, and error bars indicate 95% Wilson confidence intervals. Panels exclude respondents with missing professional position data and therefore, subgroup sample sizes may sum to one fewer than the overall valid item sample. In Figure 3 (safety and pharmaceutical awareness), responses were more heterogeneous. While 66.8% (189/283) selected severely immunocompromised patients as a high-risk group (Q14), a considerable proportion selected other options, including pregnancy in the third trimester and mild lactose intolerance. Awareness of the absence of approved fixed-dose antibiotic–probiotic combinations was moderate, with 64.5% (182/282) selecting that such formulations do not currently exist (Q15).

**Figure 3:**
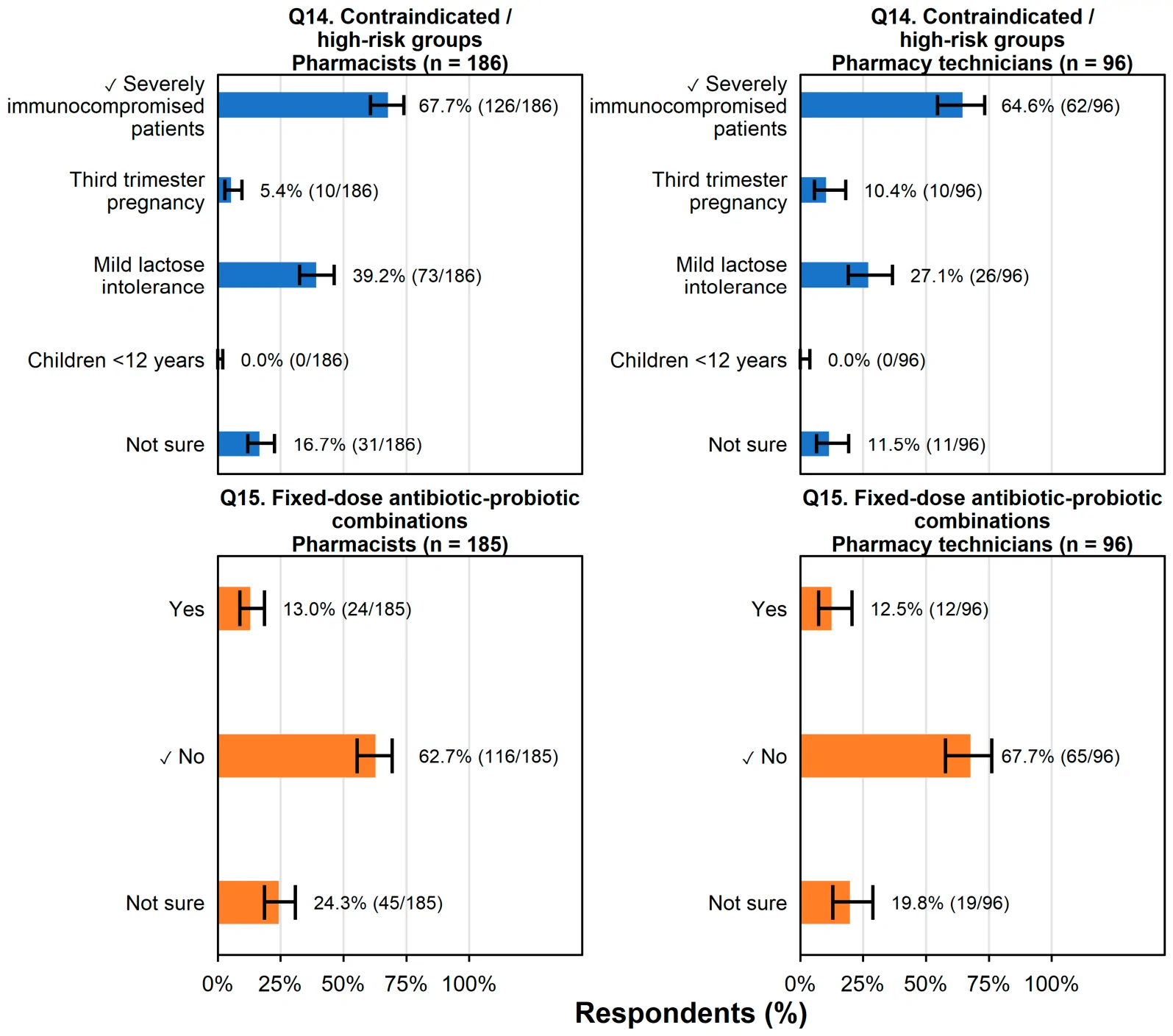
Community pharmacy professionals’ (pharmacists’ and pharmacy technicians’) responses to knowledge items regarding safety and pharmaceutical awareness of probiotics (Q14–Q15) stratified by professional position, where ✓ indicates the prespecified evidence-aligned response used in the survey scoring key. Bars represent percentages of valid responses, and error bars indicate 95% Wilson confidence intervals. For Q14, multiple responses were allowed and therefore, percentages within each professional group may exceed 100%. Panels exclude respondents with missing position data and therefore, subgroup sample sizes may sum to one fewer than the overall valid item sample.

Overall, respondents generally showed moderate to high alignment with the prespecified survey knowledge key regarding probiotics and their role in AAD, as illustrated in Figure 1, Figure 2 and Figure 3. A very high proportion of respondents selected responses that reflected that scientific evidence supports the efficacy of probiotics in AAD prevention (Q7: 269/285, 94.4%), as shown in Figure 1 (evidence and mechanisms). Similarly, 81.1% (227/280) selected the multifactorial mechanisms of probiotic action, including modulation of immune response, competition with pathogens, and production of antimicrobial substances, whereas the remaining respondents selected alternative responses or uncertainty (Q8). A similarly high proportion indicated the beneficial effect of probiotics in reducing AAD (Q9: 272/284, 95.8%).

Figure 2 presents respondents’ answers regarding the clinical use and administration of probiotics with antibiotics (Q10–Q13). Most respondents selected the prespecified appropriate timing of administration, including separation from antibiotics by 2–3 h or concurrent use with antibiotic-resistant strains (Q10: 242/282, 85.8%). However, only 34.6% (98/283) selected the response that probiotics should preferably be taken with food, depending on product characteristics (Q11). Regarding initiation of therapy, 93.7% (266/284) selected that probiotics should be started within 24–48 h after initiation of antibiotic treatment (Q12). In contrast, 57.7% (164/284) selected a 1–4-week continuation period after antibiotic therapy (Q13).

A survey composite knowledge index, calculated by summing the prespecified key responses across Q7–Q15, indicated a generally moderate-to-high level of knowledge among respondents (mean = 6.49 ± 1.48; median = 7; range: 2–9). More than half of the participants (156/285, 54.7%) were classified in the high knowledge index category, while 42.1% (120/285) had a moderate knowledge index and 3.2% (9/285) a low knowledge index.

### 3.5. Community Pharmacy Professionals’ Attitudes Toward Probiotic Use in Antibiotic Therapy

**Figure 4:**
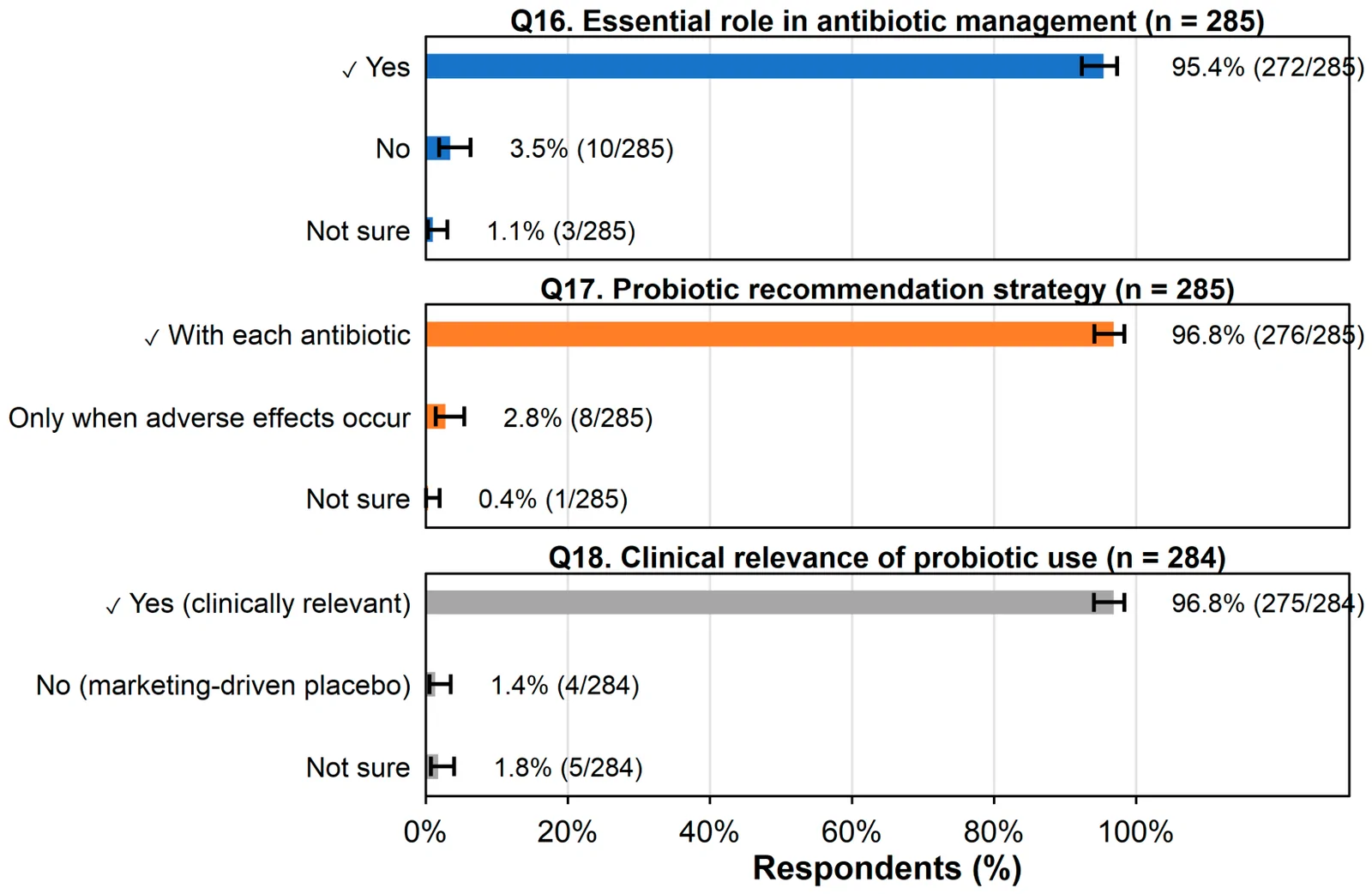
Community pharmacy professionals’ attitudes toward concomitant probiotic and antibiotic use (Q16–Q18), where ✓ indicates the response coded as more positive toward probiotic use. Bars represent percentages of valid responses, and error bars indicate 95% Wilson confidence intervals.

Overall, respondents demonstrated generally positive attitudes toward probiotic use during antibiotic therapy. As shown in Figure 4, the vast majority of participants considered that probiotic recommendation is an essential component of antibiotic management (Q16: 272/285, 95.4%), supported proactive co-recommendation with every antibiotic course (Q17: 276/285, 96.8%), and perceived probiotic use as a clinically relevant therapeutic strategy rather than a marketing-driven intervention (Q18: 275/284, 96.8%).

In addition, 200/283 (70.7%) of respondents supported the development of a single fixed-dose antibiotic–probiotic formulation (Q19), whereas 45/283 (15.9%) did not support this approach and 38/283 (13.4%) were uncertain. Regarding perceived benefits of such a formulation (Q20), 71/285 (24.9%) selected a single advantage, while the majority reported multiple benefits. The most frequently identified advantages included simplified therapy (205/285, 71.9%), improved adherence (185/285, 64.9%), better clinical outcomes (139/285, 48.8%), and improved safety (100/285, 35.1%), while 26/285 (9.1%) of respondents were uncertain. A high level of support was also observed for system-level interventions, including structured pharmaceutical counseling services during concomitant antibiotic and probiotic use (261/285, 91.6%; Q21) and the establishment of a national probiotic registry or guideline (276/284, 97.2%; Q22), including standardized information on composition (CFU), indications, and levels of clinical evidence.

The EFA-derived attitude score (Q16–Q18) ranged from 0 to 3, with a mean value of 2.89 ± 0.46, indicating generally positive attitudes toward the use of probiotics in combination with antibiotics.

### 3.6. Community Pharmacy Professionals’ Practices Related to Probiotic Recommendation and Counseling

**Figure 5:**
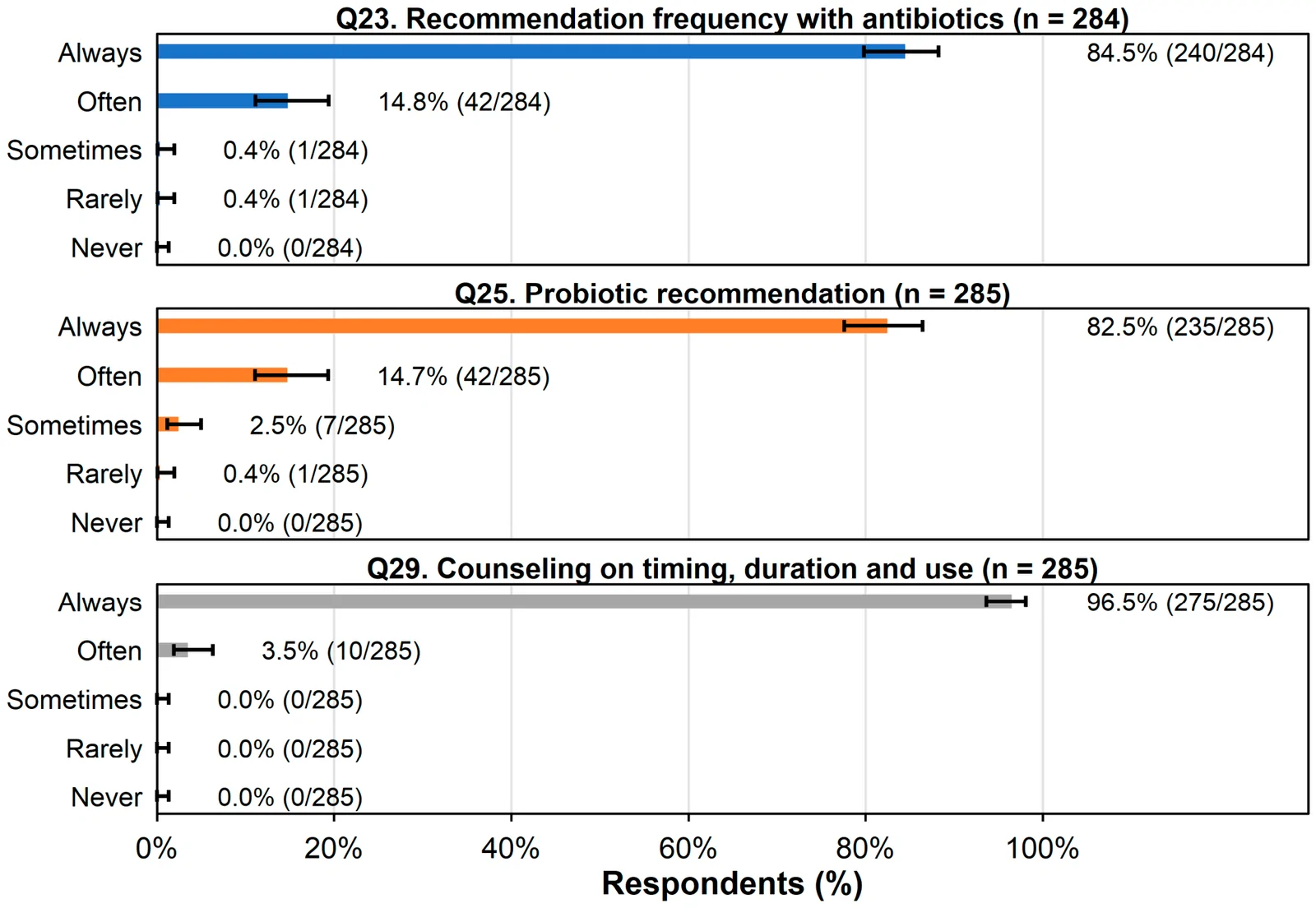
Community pharmacy professionals’ recommendation-related practice regarding concomitant probiotic and antibiotic use (Q23, Q25, Q29). Bars represent percentages of valid responses, and error bars indicate 95% Wilson confidence intervals.

**Figure 6:**
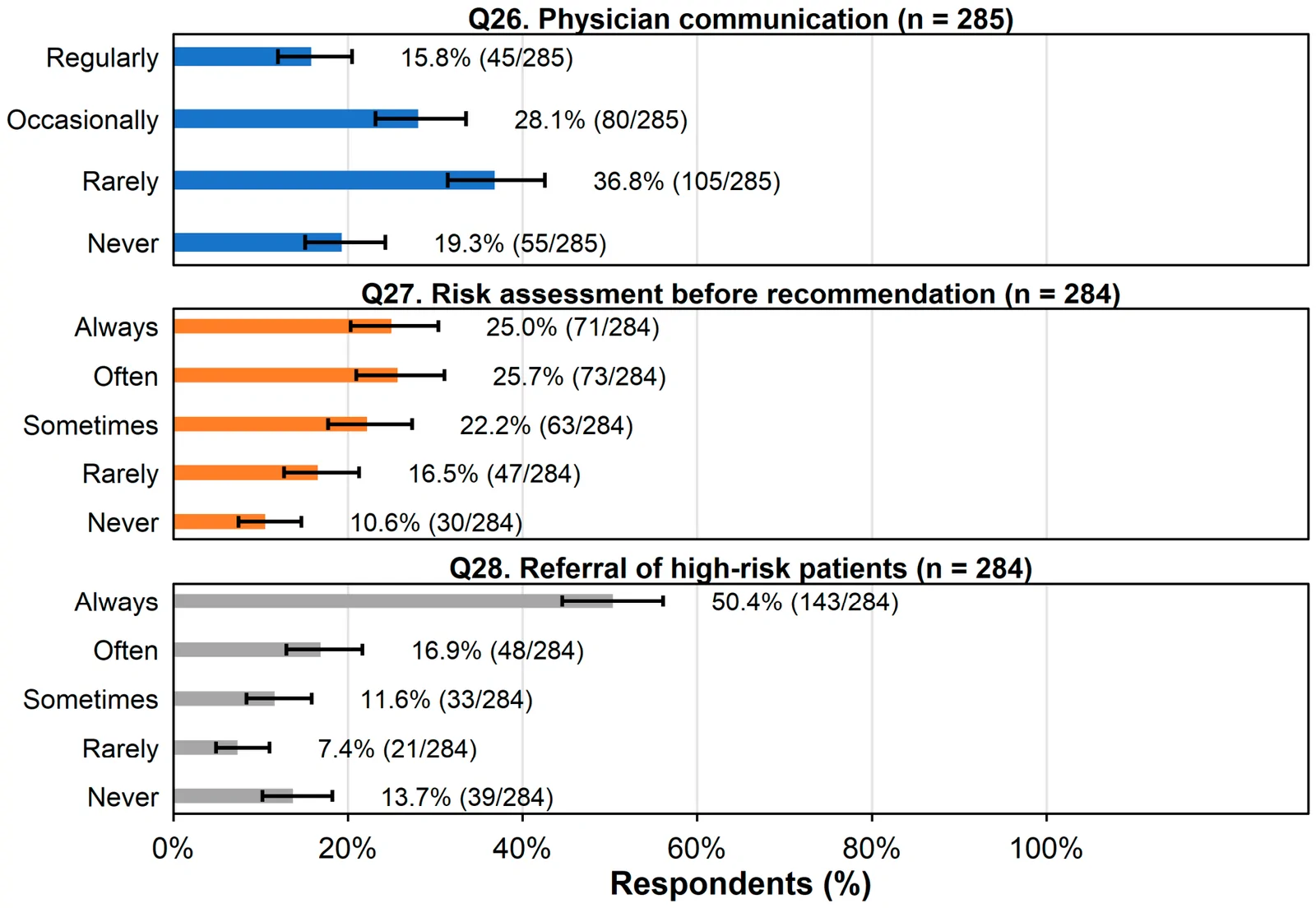
Community pharmacy professionals’ counseling-related behaviors and interprofessional practices regarding concomitant probiotics and antibiotics use (Q26–Q28). Bars represent percentages of valid responses, and error bars indicate 95% Wilson confidence intervals.

For recommendation practice-related behavior (Figure 5), most respondents reported that they always recommend probiotics together with antibiotics for AAD (Q23; 240/284, 84.5%), while 42/284 (14.8%) reported frequent recommendation. Similarly, a high proportion of respondents reported that they always recommend probiotics when not prescribed with antibiotics (Q25; 235/285, 82.5%) and always provide counseling on the timing, duration, and method of administration (Q29; 275/285, 96.5%), indicating consistently high levels of self-reported recommendation-related practice. In contrast, interprofessional communication with physicians (Q26) and patient risk assessment (Q27), as well as referral of patients at increased risk (Q28), showed more variability across respondents, as illustrated in Figure 6.

Regarding probiotic recommendations for AAD (Q24), combined multi-strain probiotic preparations were the most frequently recommended option (218/283, 77.0%), followed by *Saccharomyces boulardii* (132/283, 46.6%), *Lactobacillus* spp. (111/283, 39.2%), and *Bifidobacterium* spp. (73/283, 25.8%). Only 1/283 (0.35%) respondents reported uncertainty. As multiple responses were allowed, percentages exceed 100%. Among respondents who selected only a single option, 19/283 (6.7%) recommended exclusively *Lactobacillus* spp., 20/283 (7.1%) *Saccharomyces boulardii*, while 115/283 (40.6%) reported exclusive recommendation of combined multi-strain probiotic preparations.

The recommendation-related practice score (Q23, Q25, Q29) indicated a high frequency of self-reported recommendation behavior among respondents, with a mean score of 14.6 ± 0.95 (median = 15), ranging from 9 to 15. The counseling-related practice score (Q26–Q28) demonstrated greater variability, with a mean score of 9.59 ± 2.85 (median = 10) and observed scores ranging from 3 to 14, indicating more heterogeneous self-reported counseling behaviors among community pharmacy professionals.

The attitude and recommendation-related practice scores showed marked ceiling effects, with responses concentrated near the upper limits of the scales, resulting in restricted variability between respondents.

### 3.7. Bivariate Associations Across the KAP Domains

Following Benjamini–Hochberg false discovery rate (FDR) adjustment, categorical association analyses identified several statistically significant associations between sociodemographic variables and KAP-related outcomes (Table 2).

#### 3.7.1. Knowledge-Related Associations

Age (Q1) was significantly associated with perceived clinical effectiveness in AAD (Q9), with Cramer’s V indicating large association. However, no consistent monotonic trend was observed (Kendall’s tau-b *p* > 0.05), indicating heterogeneous, non-linear age-related difference.

Duration (years) of professional experience (Q3) was associated with perceived clinical effectiveness in AAD (Q9), with a small effect size. No consistent monotonic experience-related pattern was evident.

Professional position (Q5) was associated with responses regarding clinical effectiveness (Q9). Pharmacists showed more evidence-aligned responses compared to pharmacy technicians. Effect size indicated small association with no evidence of ordinal structure.

#### 3.7.2. Attitude-Related Associations

No attitude-related associations remained statistically significant after FDR adjustment.

#### 3.7.3. Practice-Related Associations

Gender (Q2) was associated with autonomous probiotic recommendation when a physician had not recommended it (Q25), with a small effect size. Female respondents more frequently reported autonomous recommendation, whereas male respondents showed higher proportions of conditional or less frequent engagement.

Prior education on probiotics (Q6) was associated with communication with physicians (Q26), and consideration of immunological status (Q27). While a structured trend was absent for Q26, a weak ordinal association was observed for Q27 (Kendall’s tau-b = −0.115, *p* = 0.031). Participants with prior education reported greater engagement in interdisciplinary communication and more frequent application of clinical risk considerations. In contrast, those without such training reported fragmented or inconsistent practices more often, particularly in patient risk assessment.

### 3.8. Regression Analyses of Attitudes and Practice (Recommendation and Counseling) Outcomes

Multiple linear regression analyses were conducted to examine variables associated with attitudes, recommendation, and counseling-related practice regarding probiotic use in AAD. Complete-case OLS results are presented in (Table 3). Because residual normality assumptions were not fully met, the principal coefficient estimates were additionally evaluated using robust standard errors and percentile bootstrap 95% confidence intervals based on 5000 resamples.

Across the three linear regression models, variance inflation factor (VIF) values were below 2.5, indicating no evidence of problematic multicollinearity. Durbin–Watson statistics ranged from 1.74 to 2.09, and Cook’s distance values indicated no influential observations affecting model stability. However, Shapiro–Wilk tests indicated deviations from residual normality (*p* < 0.001 for all models). Therefore, robust standard errors and percentile bootstrap 95% confidence intervals based on 5000 resamples were used as sensitivity analyses. Interpretation focused primarily on associations that were consistently supported across the conventional OLS, robust, and bootstrap analyses.

#### 3.8.1. Attitude Model

The attitude model (Q16–Q18), showed low explanatory power. No sociodemographic variables (*p* > 0.05) were significantly associated with the attitude score. The overall model remained non-significant in the robust and bootstrap sensitivity analyses.

#### 3.8.2. Recommendation-Related Practice Model

For recommendation-related practice (Q23, Q25, Q29), the model demonstrated moderate explanatory power. More favorable attitudes were associated with higher recommendation scores. Female gender was also associated with higher recommendation scores (*p* = 0.008). Both associations remained supported in the sensitivity analyses: attitude, B = 1.039, robust *p* < 0.001, bootstrap 95% CI 0.748–1.312; and gender, B = −0.445, robust *p* = 0.034, bootstrap 95% CI −0.890 to −0.047. Other variables were not significantly associated.

#### 3.8.3. Counseling-Related Practice Model

For counseling-related practice (Q26–Q28), the model showed low explanatory power. Prior education on probiotics was significantly associated with higher counseling-related practices. All other variables were not statistically significant. This association remained supported in the sensitivity analyses (B = −1.365, robust *p* < 0.001, bootstrap 95% CI −2.103 to −0.629). The attitude coefficient and some pharmacy-type contrasts differed between the conventional and sensitivity analyses and were therefore not interpreted as principal findings. Given the limited internal consistency (Cronbach’s α = 0.613) of the counseling-related practice subscale, these associations should be interpreted cautiously.

#### 3.8.4. Innovation-Related Attitudes

Ordinal logistic regression analyses were performed to examine factors associated with innovation-related perceptions regarding pharmaceutical practice (Q19, Q21, Q22) (Table 4). As previously described, all three outcomes showed a high level of acceptance in descriptive analysis, particularly for system-level interventions (Q21 and Q22). Given the limited response variability for Q21 and Q22, the descriptive distributions and previously reported bivariate analyses were considered the primary evidence of consensus, while the Q21 ordinal model was retained as an exploratory adjusted analysis. Q22 was analyzed descriptively because insufficient outcome variability prevented stable model estimation.

For Q19 (fixed-dose antibiotic–probiotic formulation), professional position was significantly associated with pharmacy technicians, showing higher odds of agreement (OR = 1.85). In addition, higher counseling-related practice scores were associated with lower odds of agreement (OR = 0.89). However, this association should be interpreted cautiously given the limited internal consistency of the counseling-related practice subscale. No other variables were significantly associated with the outcome. The model for Q21 (support for structured pharmaceutical counseling services) was not statically significant (*p* = 0.054) and no individual variable was significantly associated with the outcome. For Q22 (support for a national probiotic registry or guideline), an ordinal logistic regression model could not be estimated because near-unanimous support resulted in insufficient outcome variability.

Overall, the findings indicate a high level of acceptance of innovation-oriented pharmaceutical interventions, especially for system-level measures (Q21 and Q22). Q19 showed modest associations with professional position and counseling-related practice, whereas no robust individual associations were identified for Q21.

#### 3.8.5. Missing-Data and Multiple-Imputation Sensitivity Analyses

Little’s MCAR test for the variables included in the three final linear regression models and the ordinal regression models for Q19 and Q21 was not statistically significant, D^2^(189) = 178, *p* = 0.699, indicating no evidence against the MCAR assumption for this model-specific set of variables. Q22 was not included in this assessment because its limited outcome variability precluded stable ordinal regression estimation and it was only analyzed descriptively.

Multiple-imputation sensitivity analyses based on 20 imputed datasets were conducted for all five regression models: the three linear regression models and the ordinal regression models for Q19 and Q21. The pooled attitude model remained non-significant (R^2^ = 0.040, *p* = 0.189). Although a statistically significant gender coefficient emerged in the pooled attitude model (*p* = 0.032), it was not interpreted because the overall model remained non-significant and this coefficient was not consistently supported across the complete-case and robust analyses. The pooled recommendation model remained significant (R^2^ = 0.309, *p* < 0.001), with attitude and gender retaining statistically significant associations. The pooled counseling model also remained significant (R^2^ = 0.092, *p* = 0.002) and the association with prior education on probiotics remained supported. An isolated association with professional position emerged in the pooled counseling model (*p* = 0.022), but it was not interpreted as a robust finding because it was not consistently observed in the complete-case and robust analyses.

The pooled ordinal model for Q19 remained statistically significant overall (D1 pooled test, *p* = 0.015). The association with counseling-related practice remained statistically significant (*p* = 0.004), whereas the association with professional position was attenuated and no longer reached statistical significance after multiple imputation (*p* = 0.061). The pooled Q21 ordinal model was not statistically significant overall (D1 pooled test, *p* = 0.115), and no individual predictor was statistically significant.

The sensitivity power analysis indicated that, with *N* = 260, α = 0.05, and 80% power, the minimum detectable overall effect was partial η^2^ = 0.056 (Cohen’s f^2^ = 0.060) for the attitude model with eight degrees of freedom and partial η^2^ = 0.059 (f^2^ = 0.062) for the recommendation and counseling models with nine degrees of freedom.

The principal findings from the recommendation-related and counseling-related linear models were generally supported after multiple imputation. For Q19, the association with counseling-related practice remained supported, whereas the professional-position association was not robust to the handling of missing data. No robust adjusted associations were identified for Q21. Overall, the principal regression findings were considered robust to residual non-normality and the handling of missing data.

## 4. Discussion

### 4.1. Knowledge, Attitudes, Practices and Associated Factors

The present study included 285 community pharmacy professionals, of whom 188 were pharmacists, representing approximately 12.7% of the 1480 pharmacists employed in community pharmacies in North Macedonia (according to unpublished administrative data provided by the Pharmaceutical Chamber of North Macedonia on 24 July 2026). This indicates substantial participation from the pharmacist subgroup, but it should not be interpreted as a response rate or evidence of national representativeness because convenience sampling was used. The findings provide valuable insight into survey-based probiotic-related knowledge responses, attitudes, and self-reported practices within this professional group. The results indicate that respondents, community pharmacists and pharmacy technicians generally self-reported moderate to high alignment with the prespecified survey scoring key, highly positive attitudes and frequent self-reported recommendation practices regarding the use of probiotics for the prevention of AAD. Most respondents selected the response that probiotics may reduce AAD (95.8%) and acknowledged that supporting scientific evidence exists (94.4%). These findings are consistent with evidence from the meta-analysis showing that probiotics reduce the risk of AAD by approximately 30–40%, although efficacy is strain- and population-dependent rather than a class effect [10,28]. The knowledge items, however, did not support a unidimensional construct; therefore, the item-level findings are more informative than interpretation of the survey-based knowledge index as a single measure of factual knowledge.

Attitude responses were concentrated near the upper end of the scale, with 95.4–96.8% selecting the more favorable options. Such high levels exceed those reported in previous studies among healthcare professionals, where attitudes are generally positive but more variable due to uncertainty regarding strain-specific evidence and guideline familiarity [29,30,31]. However, the finding that 96.8% favored recommending a probiotic with every antibiotic course should be interpreted as a strong pro-probiotic orientation rather than as evidence of optimal practice. Current guidelines evaluate specific strains or defined combinations at studied doses for particular populations and indications, but they do not support assuming that all probiotic products are interchangeable or necessary with every antibiotic course [19]. The marked ceiling effect may also have limited differentiation in the adjusted attitude model, which was not statistically significant. By contrast, higher attitude scores were associated with higher recommendation-related practice scores. This high acceptance may reflect increasing integration of microbiome-related concepts into pharmacy practice and expanding exposure to probiotic education. Its coexistence with high self-reported recommendation rates is consistent with an association between attitudes and behavior [32], although causal direction cannot be inferred from this cross-sectional study.

Despite these positive findings, a notable gap was observed between general awareness and practical clinical knowledge with the prespecified survey scoring key. Only 34.6% of respondents selected the prespecified response regarding the influence of food intake on probiotic administration, while 57.7% selected the prespecified proposed duration of 1–4 weeks as the appropriate time of use after antibiotic therapy. In addition, only 66.8% identified immunocompromised patients as a high-risk group. These findings indicate that community pharmacy professionals were familiar with the general therapeutic role of probiotics but showed less consistent alignment with the prespecified survey key regarding clinically relevant application details, particularly in vulnerable populations and administration conditions. Similar findings have been reported internationally among healthcare professionals, particularly regarding timing of administration and safety considerations [14]. After FDR adjustment, age, professional experience, and professional position were associated with recognition of clinical effectiveness, but the patterns were non-linear and did not support a simple demographic gradient. The safety finding remains clinically important because vulnerable patients may have a greater risk of invasive infection and require individualized assessment or medical referral [33].

A strong preference for combined multi-strain probiotic preparations was observed for the management of AAD (77.0%), while single-strain recommendations were less frequent, indicating a predominant inclination toward multi-strain formulations in clinical practice. This preference should not be interpreted as evidence that multi-strain products are generally superior to single-strain products, considering that efficacy remains formulation-, population- and indication-specific [34]. Accordingly, some clinical recommendations acknowledge the potential benefits of multi-strain formulations, although they also emphasize that efficacy remains strain- and combination-specific, underscoring the need for careful selection based on evidence-based formulations rather than generic probiotic categories [35,36]. The result therefore highlights the need for product-specific counseling and for clear communication of the evidence supporting the particular preparation being recommended.

The practice findings also distinguish frequent recommendation from more complex counseling behavior. Recommendation-related responses were concentrated near the scale maximum, whereas communication with physicians, risk assessment and referral showed substantially greater variability. This distinction was supported by the psychometric analyses, which identified recommendation-related and counseling-related practice as two weakly correlated dimensions. In the adjusted recommendation model, more positive attitudes and female gender were associated with higher recommendation scores and these associations were generally retained in robust and multiple-imputation analyses. These findings suggest that both individual-level and organizational factors are associated with community pharmacy professionals’ self-reported behavior, indicating that clinical decision-making regarding probiotics might be linked to community pharmacy professionals’ perceptions and workplace context rather than knowledge alone [37]. However, the observed gender association should be interpreted cautiously, as unmeasured professional or workplace-related factors may have contributed to this difference. Furthermore, a composite knowledge predictor was not included in the final adjusted practice models because the knowledge items did not support a sufficiently reliable unidimensional scale.

Recent continuous professional education was associated with self-reported counseling-related practice. Community pharmacy professionals who had recent education on probiotics self-reported significantly higher counseling practices, particularly in risk assessment and interdisciplinary communication. This finding does not demonstrate a causal educational effect, but it supports the practical relevance of targeted continuing education focused on risk identification, product-specific use, referral and interprofessional communication. This aligns with previous findings showing that targeted continuous education improves community pharmacy professionals’ confidence [38] and quality of patient counseling [39] across therapeutic domains. Prospective or controlled studies are needed to determine whether targeted continuing education will improve counseling quality in the North Macedonia context.

The counseling model nevertheless explained only a small proportion of the observed variance (R^2^ = 0.097, adjusted R^2^ = 0.064), and the counseling subscale had limited internal consistency. Pharmacy-type contrasts were not consistent across the conventional and sensitivity analyses and were therefore not interpreted as principal findings. Consequently, the data do not support firm conclusions that counseling is systematically stronger in one pharmacy setting than another. Other unmeasured organizational and interpersonal factors, such as prescription volume, staffing, workload, pharmacy policies and opportunities for communication with physicians, may account for additional variation in counseling practices and should be examined in future studies [40].

Finally, there was strong support among respondents for system-level improvements, including structured counseling services, development of fixed-dose antibiotic–probiotic formulations, and a national probiotic guideline. These findings indicate a clear perceived need for standardization and improved clinical infrastructure in probiotic use. However, regression analysis revealed limited explanatory power of the analyzed variables. Only the Q19 (fixed-dose antibiotic–probiotic formulation) outcome had a significant complete-case model. Counseling-related practice remained associated with Q19 after multiple imputation, whereas the professional-position association attenuated and was no longer statistically significant. No significant variables were identified for structured counseling services (Q21), while national guideline development (Q22) could not be modeled. This discrepancy between high descriptive support and weak predictive modeling suggests that these system-level preferences may reflect broadly shared professional expectations rather than being driven by specific sociodemographic or practice-related factors.

### 4.2. Strengths and Limitations

This study is limited by its cross-sectional design, which precludes causal inference, and by potential self-reporting bias. The high proportion of “always” responses for several practice-related items may also reflect social desirability bias rather than actual routine practice. Ceiling effects in the attitude and recommendation-related practice outcomes restricted variability and may have reduced the ability to detect associations; these findings should therefore be interpreted as broadly favorable self-reports rather than precise differences between respondents. Future studies should incorporate standardized clinical patient vignettes or objective practice assessments to evaluate applied decision-making and counseling performance more accurately. Because the questionnaire was self-administered, it was not possible to determine whether participants consulted external information sources while completing it, which may have influenced and potentially increased the observed survey-based knowledge index values. Several questionnaire items simplified clinically conditional issues into a single keyed response classified according to the prespecified survey scoring. In particular, Q10–Q13 did not fully capture variability by strain, defined combination, dose, product formulation and labeling, population, indication, antibiotic susceptibility, and clinical context. Likewise, Q16–Q18 and the recommendation-related practice items measured favorability and frequency rather than verified appropriateness. The dichotomous scoring approach may therefore misclassify nuanced clinical reasoning and may overstate the extent of guideline-concordant knowledge or practice. The borderline KMO value for the practice domain, retention of the second practice factor despite not meeting the eigenvalue criterion and limited internal consistency of the counseling-related practice subscale (Cronbach’s α = 0.613) warrant cautious interpretation of analyses involving this construct. In addition, the post hoc CFA used the same sample as the EFA and therefore does not constitute independent structural validation. The limited explanatory power of some regression models, particularly the counseling-related practice model, suggests that relevant organizational, interpersonal and workplace-related factors, such as prescription volume, staffing, workload and time available for counseling, were not fully captured. Although women predominated in the sample, the sex distribution among pharmacists closely matched the national register; however, comparable national data were unavailable for pharmacy technicians. The sample also included a higher proportion of respondents from large pharmacy chains than indicated by the national distribution of community pharmacy premises. Because national data on the number of pharmacists and pharmacy technicians employed by pharmacy type were unavailable, valid person-level post-stratification weights could not be constructed. This may limit the national generalizability of the findings. In addition, convenience sampling and the unknown number of professionals who received or viewed the survey invitation prevented calculation of a response rate and may further limit the representativeness of the findings. Recruitment through professional networks may have introduced selection bias toward more active or affiliated professionals. Nevertheless, the study provides valuable and comprehensive insight into survey-based knowledge responses, attitudes and self-reported practices among community pharmacy professionals in North Macedonia, using questionnaire domains that underwent internal psychometric evaluation.

### 4.3. Implications

The findings may help define priorities for future professional education and policy development regarding probiotic use in community pharmacy practice in North Macedonia. Continuing professional education could focus particularly on areas in which responses showed lower alignment with the prespecified survey scoring key or greater variability, including administration conditions, duration of use, safety in vulnerable populations, patient risk assessment, referral of high-risk patients and communication with physicians. Educational content should emphasize that probiotic recommendations are dependent on the specific strain or combination, product, dose, population, indication, and clinical context.

The strong respondent support for structured pharmaceutical counseling services and a national probiotic guideline suggests that such initiatives may be acceptable to community pharmacy professionals. This provides a basis for considering the development of nationally adapted educational resources, counseling frameworks, and evidence-based guidelines. However, these initiatives should initially be implemented and evaluated as prospective interventions using objective measures of counseling quality and professional practice before conclusions are drawn regarding their effects on clinical practice or patient outcomes.

## 5. Conclusions

Community pharmacy professionals generally demonstrated moderate-to-high alignment with the prespecified survey scoring key and positive attitudes toward probiotic use in the prevention of antibiotic-associated diarrhea, accompanied by generally frequent self-reported recommendation practices. However, important gaps remain in specific clinical knowledge, particularly regarding administration, duration of use and patient safety considerations, as well as variability in counseling behaviors. Because probiotic recommendations depend on the strain or defined combination, product, population, indication, and clinical context, these findings should be interpreted as responses to survey-specific, evidence-aligned benchmarks rather than verification of universally applicable clinical rules. More favorable attitudes were associated with higher recommendation-related practice scores. The independent association of the survey-based knowledge index with practice could not be tested because the knowledge items did not support a reliable unidimensional score. Strengthening continuous professional education to address the identified gaps and implementing standardized, evidence-based national guidelines (including strain, product, population and indication-specific recommendations) in response to strong support expressed by respondents may help bridge the gap between positive perceptions and optimal clinical practice, with potential to improve the quality and consistency of probiotic-related pharmaceutical care. The effectiveness of these measures should be evaluated in future studies.

## Figures and Tables

**Table 1:** Demographic characteristics of the studied population.

Variable	Category	N (%) *
Total		285
Gender	Male	29 (10.2)
	Female	252 (88.4)
	Not important	/
	Missing	4 (1.4)
Age	18–24	5 (1.8)
	25–44	161 (56.5)
	45–59	98 (34.4)
	60–74	9 (3.2)
	75+	/
	Missing	12 (4.2)
Professional experience (years)	≤5	33 (11.6)
	6–10	55 (19.3)
	11–20	96 (33.7)
	>20	92 (32.3)
	Missing	9 (3.2)
Community pharmacy type	Independent (private) pharmacy	56 (19.6)
	Small pharmacy chain (2–5 pharmacies)	33 (11.6)
	Medium-sized pharmacy chain (6–20 pharmacies)	27 (9.5)
	Large pharmacy chain (more than 20 pharmacies)	165 (57.9)
	Missing	4 (1.4)
Professional position	Pharmacist	188 (66)
	Pharmacy technician	96 (33.7)
	Missing	1 (0.4)
Continuous education on probiotics in the last 2 years	Yes	172 (60.4)
	No	113 (39.6)
	Missing	/

* Percentages may exceed 100% due to rounding.

**Table 2:** Associations between community pharmacy professionals’ sociodemographic variables and knowledge items, attitude, and practice outcomes.

Variable	Outcome (Item/Domain)	Test/Statistic *	*p*-Value	FDR-Adjusted *p*-Value	Cramer’s V
Age (Q1)	Clinical effectiveness in AAD (Q9)	FFH exact (Monte Carlo)	0.001	0.035	0.309
Gender (Q2)	Probiotic recommendation (Q25)	FFH exact (Monte Carlo)	0.001	0.035	0.268
Experience (Q3)	Clinical effectiveness in AAD (Q9)	FFH exact (Monte Carlo)	0.001	0.035	0.164
Position (Q5)	Clinical effectiveness in AAD (Q9)	FFH exact (Monte Carlo)	<0.001	0.021	0.244
Education (Q6)	Physician communication (Q26)	Pearson χ^2^(3) = 18.5	<0.001	0.021	0.255
	Risk assessment before recommendation (Q27)	Pearson χ^2^(4) = 17.2	0.002	0.036	0.246

* Pearson’s chi-square test was used when expected-count assumptions were met; otherwise, the Fisher–Freeman–Halton (FFH) exact test with Monte Carlo simulation (B = 200,000) was applied. *p*-values were adjusted collectively across 126 bivariate association tests using the Benjamini–Hochberg false discovery rate procedure. Only associations with an FDR-adjusted *p*-value < 0.05 are presented.

**Table 3:** Complete-case OLS regression models of factors associated with community pharmacy professionals’ attitudes and recommendation- and counseling-related practices.

Outcome	Model Fit	Significant Associations	β *	*p*-Value
Attitude (Q16–Q18)	F(8, 251) = 1.3, *p* = 0.243; R^2^ = 0.04; Adj R^2^ = 0.009	None, model not significant		
Recommendation (Q23,25,29)	F(9, 250) = 13.3, *p* < 0.001; R^2^ = 0.324; Adj R^2^ = 0.300	Attitude	0.512	<0.001
		Gender (male vs. female)	−0.458	0.008
Counseling (Q26–Q28)	F(9, 250) = 2.97, *p* = 0.002; R^2^ = 0.097; Adj R^2^ = 0.064	Education on probiotics No vs. Yes	−0.480	<0.001

* β denotes the standardized OLS regression coefficient. Only statistically significant associations from the complete-case OLS models are shown.

**Table 4:** Ordinal logistic regression analysis of innovation-related perceptions of community pharmacy professionals (Q19, Q21 and Q22).

Outcome	Model Fit	Significant Associations	β	*p*-Value
Q19—Fixed-dose antibiotic–probiotic formulation	χ^2^(11) = 25.4, *p* = 0.008; Nagelkerke R^2^ = 0.078	Professional position (pharmacy technicians vs. pharmacists)	0.616	0.045
		Counseling-related practice	−0.121	0.019
Q21—Structured pharmaceutical counseling services	χ^2^(11) = 19.4, *p* = 0.054; Nagelkerke R^2^ = 0.115	None, overall model not statistically significant	/	/
Q22—National probiotic registry/guideline	Not estimated due to insufficient outcome variability	Not applicable	/	/

## Data Availability

Processed datasets and analysis generated during the study are available from the corresponding author upon reasonable request.

## Supplementary Materials

The following supporting information can be downloaded at: https://www.mdpi.com/article/10.3390/healthcare14162602/s1. Questionnaire: Community Pharmacy Professionals’ Attitudes, Knowledge, and Practices Regarding the Combined Use of Antibiotics and Probiotics; Table S1: Valid and missing responses by questionnaire item (N = 285).
