# Supplementary material for: Use of Probiotics with Antibiotics: Knowledge, Attitudes, and Practices in Community Pharmacy
Source: Healthcare (Basel). 2026 Aug 19;14(16):2602. doi: 10.3390/healthcare14162602 (PMC13512189; doi:10.3390/healthcare14162602)
Supplement: Supplementary file 1 [file healthcare-14-02602-s001.zip › healthcare-4411923-supplementary.pdf]

Supplementary Material

Questionnaire: Community Pharmacy Professionals' Attitudes, Knowledge, and Practices Regarding the Combined Use of Antibiotics and Probiotics

English translation of the original Macedonian-language questionnaire administered in the study

Dear colleagues, this questionnaire aims to assess pharmacists' knowledge, attitudes, and practices regarding the concomitant use of antibiotics and probiotics. The collected data will contribute to a better understanding of current pharmacy practice and help identify needs for improving education and patient counseling in this area. The questionnaire is anonymous, and all responses will be used exclusively for scientific research purposes. The questionnaire consists of 29 questions and requires approximately 5–7 minutes to complete. Thank you for your participation and contribution

I. Demographic and Professional Characteristics

1. Age: __________

2. Gender:

A. Female
B. Male
C. Prefer not to disclose

3. Years of professional experience: __________

4. Type of community pharmacy:

A. Independent (privately owned) pharmacy
B. Small pharmacy chain (2–5 pharmacies)
C. Medium-sized pharmacy chain (6–20 pharmacies)
D. Large pharmacy chain (more than 20 pharmacies)

5. Professional position:

A. Pharmacist
B. Pharmacy technician

6. Have you participated in any training or continuing professional education on probiotics during the past two years?

A. Yes
B. No

II. Knowledge

7. Is there scientific evidence supporting the effectiveness of probiotics in preventing antibiotic-associated diarrhea (AAD)?

A. Yes
B. No
C. Not sure

8. Which of the following best describes the mechanisms of action of probiotics?

A. Modulation of the immune response, competition with pathogenic microorganisms, and production of antimicrobial substances
B. Enhancement of the intestinal mucosal barrier as the sole mechanism of action
C. Systemic absorption and direct antimicrobial activity in the bloodstream
D. Reduction of gastric acidity as the primary mechanism of action
E. Not sure

9. What do clinical studies indicate regarding the effect of probiotics on AAD?

A. They reduce its incidence
B. They have no significant effect
C. They increase its incidence
D. Not sure

10. What is the recommended time interval between administering an antibiotic and a probiotic?

A. No interval is required
B. One hour
C. Two to three hours; however, they may be taken concomitantly if the probiotic is resistant to the antibiotic
D. Not sure

11. What is the most appropriate way to administer an antibiotic and a probiotic in relation to meals?

A. The probiotic on an empty stomach, and the antibiotic according to its prescribing information
B. Both on an empty stomach
C. Both with food
D. The probiotic with food and the antibiotic according to its prescribing information
E. Not sure

12. When should probiotic therapy ideally be initiated to prevent AAD?

A. After completion of antibiotic therapy
B. Within 24–48 hours of initiating antibiotic therapy
C. At least 5 days after the first antibiotic dose
D. Only after diarrhea develops
E. Not sure

13. For how long should probiotic use be continued after completion of antibiotic therapy?

A. Stop immediately after completing antibiotic therapy
B. Continue for 1–2 days
C. Continue for 1–4 weeks
D. Continue for 6 months
E. Not sure

14. Which of the following patient groups is at increased risk from probiotic use or has contraindications to probiotic therapy?

A. Pregnant women in the third trimester
B. Severely immunosuppressed patients
C. Individuals with mild lactose intolerance
D. Children younger than 12 years
E. Not sure

15. Are any approved fixed-dose formulations containing both an antibiotic and a probiotic currently available in clinical practice?

A. Yes
B. No
C. Not sure

III. Attitudes

16. Do you consider recommending a probiotic to be an essential component of antibiotic therapy?

A. Yes
B. No
C. Not sure

17. In your opinion, should probiotic be recommended routinely with every antibiotic prescription or only when patients experience antibiotic-related adverse effects?

A. With every antibiotic prescription
B. Only when adverse effects occur
C. Not sure

18. Do you consider the concomitant use of probiotics with antibiotics to be clinically beneficial or primarily marketing-driven intervention with limited clinical value?

A. Clinically beneficial
B. Primarily a marketing-driven intervention
C. Not sure

19. Would you support the availability of a single dosage form combining an antibiotic and a probiotic?

A. Yes
B. No
C. Not sure

20. What advantages would you expect from a single dosage form combining an antibiotic and a probiotic? *(Select all that apply)*

A. Improved treatment adherence
B. Improved safety
C. Simplified treatment
D. Improved clinical outcomes
E. Not sure

21. Would you support the introduction of structured pharmaceutical counseling services for patients receiving concomitant antibiotic and probiotic therapy?

A. Yes
B. No
C. Not sure

22. Do you think a publicly accessible national probiotic registry or guideline should be developed, including information on probiotic composition (CFU), approved indications, and the available clinical evidence supporting efficacy (e.g., meta-analyses and clinical practice guidelines)?

A. Yes
B. No
C. Not sure

IV. Practice

23. How often do you recommend a probiotic together with an antibiotic to prevent AAD?

A. Always
B. Often
C. Sometimes
D. Rarely
E. Never

24. Which probiotics do you most frequently recommend for preventing AAD? *(Select all that apply)*

A. *Lactobacillus* spp.
B. *Saccharomyces boulardii*
C. *Bifidobacterium* spp.
D. Multi-strain probiotic preparations
E. Not sure

25. If a physician has NOT prescribed a probiotic together with an antibiotic, how often do you recommend one?

A. Always
B. Often
C. Sometimes
D. Rarely
E. Never

26. How often do you communicate with physicians (including family physicians) regarding the concomitant use of antibiotics and probiotics?

A. Regularly
B. Occasionally
C. Rarely
D. Never

27. Before recommending a probiotic, how often do you ask patients about their immune status, gastrointestinal disorders, recent abdominal surgery, or the presence of indwelling medical devices (e.g., a central venous catheter, peripherally inserted central catheter, or prosthetic heart valve)?

A. Always
B. Often
C. Sometimes
D. Rarely
E. Never

28. When you identify a patient who may be at increased risk from probiotic use, how often do you refer the patient to a physician (including a family physician)?

A. Always
B. Often
C. Sometimes
D. Rarely
E. I do not routinely assess patients for probiotic-related risk

29. When dispensing both an antibiotic and a probiotic, how often do you counsel patients on the recommended timing, duration, and method of administration?

A. Always
B. Often
C. Sometimes
D. Rarely
E. Never

Supplementary Table S1. Valid and missing responses by questionnaire item (N = 285)

Item	Questionnaire item	Valid responses, n	Missing responses, n (%)
Q1	Age	273	12 (4.2)
Q2	Gender	281	4 (1.4)
Q3	Years of professional experience	276	9 (3.2)
Q4	Type of community pharmacy	281	4 (1.4)
Q5	Professional position	284	1 (0.4)
Q6	Probiotic-related continuing education during the previous two years	285	0 (0.0)
Q7	Scientific evidence supporting probiotics for AAD prevention	285	0 (0.0)
Q8	Mechanisms of probiotic action	280	5 (1.8)
Q9	Clinical effectiveness of probiotics in AAD	284	1 (0.4)
Q10	Recommended interval between antibiotic and probiotic administration	282	3 (1.1)
Q11	Administration of antibiotics and probiotics in relation to food intake	283	2 (0.7)
Q12	Timing of probiotic treatment initiation	284	1 (0.4)
Q13	Duration of probiotic use after antibiotic therapy	284	1 (0.4)
Q14	Patient groups at risk or with contraindications for probiotic use*	283	2 (0.7)
Q15	Availability of approved fixed-dose antibiotic–probiotic formulations	282	3 (1.1)
Q16	Probiotic recommendation as an essential part of antibiotic treatment	285	0 (0.0)
Q17	Proactive probiotic recommendation strategy	285	0 (0.0)
Q18	Clinical relevance of concomitant probiotic use	284	1 (0.4)
Q19	Support for a fixed-dose antibiotic–probiotic formulation	283	2 (0.7)
Q20	Expected benefits of a fixed-dose antibiotic–probiotic formulation*	285	0 (0.0)
Q21	Support for structured pharmaceutical counseling services	285	0 (0.0)
Q22	Support for a national probiotic registry or guideline	284	1 (0.4)
Q23	Frequency of probiotic recommendation with antibiotics	284	1 (0.4)
Q24	Probiotics most frequently recommended for AAD*	283	2 (0.7)
Q25	Probiotic recommendation when not prescribed by a physician	285	0 (0.0)

Q26	Communication with physicians regarding combined antibiotic–probiotic use	285	0 (0.0)
Q27	Patient risk assessment before probiotic recommendation	284	1 (0.4)
Q28	Referral of at-risk patients to a physician	284	1 (0.4)
Q29	Counseling on timing, duration, and administration	285	0 (0.0)
